# Field efficacy and safety of a novel oral chewable tablet containing sarolaner, moxidectin and pyrantel (Simparica Trio™) against naturally acquired gastrointestinal nematode infections in dogs presented as veterinary patients in Europe and the USA

**DOI:** 10.1186/s13071-020-3947-0

**Published:** 2020-03-01

**Authors:** Csilla Becskei, Kristina Kryda, Daphne Fias, Stacey L. Follis, Magda Wozniakiewicz, Sean P. Mahabir, Robert Farkas

**Affiliations:** 1 Veterinary Medicine Research and Development, Zoetis, Mercuriusstraat 20, 1930 Zaventem, Belgium; 2 Veterinary Medicine Research and Development, Zoetis, 333 Portage St., Kalamazoo, MI 49007 USA; 3 Department of Parasitology and Zoology, University of Veterinary Medicine, István u. 2, 1078 Budapest, Hungary

**Keywords:** *Ancylostoma caninum*, Ascarids, Hookworms, Nematodes, Roundworms, Simparica Trio™, *Toxascaris leonina*, *Toxocara canis*, *Uncinaria stenocephala*

## Abstract

**Background:**

Gastrointestinal nematodes are parasites that commonly infect dogs, and infections can be subclinical or may cause considerable clinical disease. Some species are zoonotic and may also cause clinical disease in humans. Year-round treatment of dogs is recommended to eliminate existing infections, which also indirectly reduces the potential for subsequent human exposure to zoonotic species. Here we present two studies that evaluated the safety and efficacy of a novel chewable oral tablet containing sarolaner, moxidectin and pyrantel against gastrointestinal nematode infections in dogs presented as veterinary patients in Europe and the USA.

**Methods:**

Dogs naturally infected with *Toxocara canis*, *Toxascaris leonina*, *Ancylostoma caninum* and/or *Uncinaria stenocephala* were enrolled in the European study, and dogs naturally infected with *T. canis* were enrolled in the USA study. The animals were treated once orally with Simparica Trio™ tablets to provide 1.2–2.4 mg/kg sarolaner, 24–48 µg/kg moxidectin and 5–10 mg/kg pyrantel (as pamoate salt) or with a commercially available product according to the label directions as positive control. Efficacy was based on the post-treatment reduction in geometric mean egg counts (per gram feces) 7 or 10 days after treatment compared to pre-treatment egg counts.

**Results:**

Simparica Trio™ was well tolerated in both studies. In the European study, geometric mean egg counts for *T. canis*, *T. leonina*, *A. caninum* and *U. stenocephala* were reduced by ≥ 98.3% in the Simparica Trio™ group and by ≥ 97.4% in the afoxolaner + milbemycin oxime group. In the USA study, geometric mean egg counts for *T. canis* were reduced by 99.2% in the Simparica Trio™ group and by 98.6% in the ivermectin + pyrantel group. In the USA study, 48 and 10 dogs in the Simparica Trio™ and the ivermectin + pyrantel group, respectively, were co-infected with *A. caninum* and the reduction in the post-treatment mean fecal egg counts were 98.6% and 74.7%, respectively.

**Conclusions:**

A single oral administration of Simparica Trio™ chewable tablets was well tolerated and was effective in the treatment of dogs with naturally occurring gastrointestinal nematode infections presented as veterinary patients in Europe and the USA.

## Background

Gastrointestinal nematodes are parasites that commonly infect dogs worldwide. Infections can be subclinical or may cause considerable clinical disease, and even death in severe cases. Two of the most common gastrointestinal nematodes infecting dogs, *Toxocara canis* (canine roundworm) and *Ancylostoma caninum* (canine hookworm) are zoonotic, and infections in humans can cause significant clinical disease, especially in young children [1, 2]. Year-round monthly treatment of dogs with anthelmintics is recommended to eliminate existing gastrointestinal infections, thereby decreasing environmental contamination and reducing the risk for human exposure [3, 4]. A variety of approved medications are currently available for use in dogs [3, 4]. A novel chewable oral tablet (Simparica Trio™, Zoetis, Parsippany, NJ, USA) containing sarolaner, moxidectin and pyrantel was recently shown in laboratory studies to be ≥ 95.2% effective against immature adult (L5) and adult *T. canis*, and ≥ 98.4% effective against L4 larval, immature adult (L5), and adult *A. caninum* in dogs with experimental infections [5, 6]. Here we present two studies in which the safety and efficacy of Simparica Trio™ was evaluated against naturally occurring gastrointestinal nematode infections in dogs presented as veterinary patients in Europe and the USA.

## Methods

Masked, randomized, positive-controlled, multi-center field studies were conducted in Europe and the USA to evaluate the efficacy and safety of a single oral dose of Simparica Trio™ against naturally occurring nematode infections in dogs presented as veterinary patients. In the European study, efficacy was evaluated against ascarid (*T. canis* and *T. leonina*) and hookworm (*A. caninum* and *U. stenocephala*) infections in dogs enrolled from 45 participating veterinary practices in Germany (10 sites), Hungary (20 sites) and Portugal (15 sites). In the USA study, efficacy was evaluated against *T. canis* infections in dogs enrolled from 18 participating veterinary practices from 14 different states located in various geographical locations including the Northeast (Connecticut, New York, Pennsylvania), Southeast (Louisiana, Maryland, Tennessee,Virginia), West (Colorado, Oregon), Midwest (Illinois, Michigan, Missouri) and Southwest (Oklahoma, Texas). In most states only single clinics enrolled cases, except for Texas and Pennsylvania where 3 and 2 clinics respectively, were involved in the study.

Both studies were conducted in accordance with the World Association for the Advancement of Veterinary Parasitology (WAAVP) guidelines for evaluating the efficacy of anthelmintics for dogs and cats [7] and complied with Good Clinical Practices [8]. All personnel involved with the collection of efficacy and safety data were masked to treatment. Treatments were administered by the dog owner or unmasked study personnel who were not involved in masked study activities.

### Animals

The patient population was recruited from dogs at least 8 weeks of age and at least 1.3 kg body weight which corresponded to the smallest weight that could be dosed with a single, whole tablet. There were no breed or sex restrictions, however pregnant females or those intended for breeding were not eligible for enrollment. Dogs living in or with a history of travel to heartworm endemic regions were confirmed to be negative for heartworm (*Dirofilaria immitis*) antigen using commercially available test kits and for microfilaria using blood smear, filter test or Knott’s test according to each clinics’ standard procedure. Enrolled dogs had not received treatment with any oral or topically applied product with efficacy against gastrointestinal nematodes within the 10 days prior to study treatment and had not received injectable moxidectin (Guardian® Elanco, Greenfield, IN, USA/ Proheart® 6, Zoetis Parsippany, NJ, USA) within the 6 months prior to study treatment. Enrolled dogs were confirmed to be infected by detection of one or more eggs of the target nematode(s) per gram of feces by quantitative fecal examination.

In the European study, enrolled dogs were infected with *T. canis*, *T. leonina*, *A. caninum*, and/or *U. stenocephala.* Enrollment was restricted to households with a maximum of three dogs. In multiple dog households, the dog infected with the most nematodes of interest or with the highest fecal egg count was selected as the primary patient, and all other dogs in the household were considered supplementary patients.

In the USA study, dogs were infected with *T. canis* and enrollment was limited to only one dog from each household. If more than one dog was eligible in the household, the animal whose name began with the letter that came first alphabetically was selected for enrollment.

### Quantitative fecal egg counts

Quantitative fecal egg counts were conducted on fecal samples collected within 7 days prior to study treatment, and at 7 (European study) or 10 days (USA study) post-treatment. Feces were collected from a recently voided sample or directly from the dog and stored refrigerated prior to shipment on the same day or the following day to a central laboratory (University of Veterinary Medicine, Department of Parasitology and Zoology, Budapest, Hungary for the European study; TRS Labs, Inc, Athens, GA, USA for the USA study) for identification and enumeration of the nematode eggs present. The number of nematode eggs per gram of feces was determined using a quantitative centrifugation-flotation technique with a sensitivity of 1 egg/gram of feces. Eggs were identified by personnel with training and experience to accurately identify nematode eggs using known morphologic characteristics [9].

### Randomization

Dogs were allocated to one of the two treatment groups according to a pre-determined generalized, randomized block design with a one-way treatment structure replicated in multiple clinics. At each clinic, dogs were blocked based on order of enrollment. In the European study, primary dogs were blocked in groups of three and allocated randomly to treatment with sarolaner + moxidectin + pyrantel (Simparica Trio™) or the positive control, afoxolaner + milbemycin oxime (NexGard® Spectra; Boehringer-Ingelheim, Ingelheim, Germany) in a 2:1 ratio. Any supplementary dogs received the same treatment as the primary dog in the same household. In the USA study, dogs were blocked in groups of four and allocated randomly to treatment with sarolaner + moxidectin + pyrantel (Simparica Trio™) or the positive control, ivermectin + pyrantel pamoate (Heartgard® Plus; Boehringer-Ingelheim, Ingelheim, Germany) in a 3:1 ratio.

### Treatment

Simparica Trio™ chewable tablets were provided in six different tablet strengths to provide dose ranges of 1.2–2.4 mg/kg sarolaner, 24–48 µg/kg moxidectin and 5–10 mg/kg pyrantel (as pamoate salt). The positive control products were dosed according to their approved commercial dosing instructions.

Treatments were administered on Day 0 by the dog owner or an unmasked study participant. Dose was determined based on the body weight collected on Day 0. There were no restrictions regarding the prandial state at the time of treatment administration; therefore, tablets could be administered with or without food. Tablets could be offered free choice by hand or administered directly by ‘pilling’. Dogs were observed for several minutes after dose administration to ensure that the entire dose was consumed.

### Safety assessments

Physical examinations were performed by a veterinarian on all dogs prior to treatment administration and again at the post-treatment visit, and at any unscheduled clinic visit that occurred during the study period. Any abnormal clinical signs observed by the veterinarians or the dog owner was recorded, as were any concomitantly administered medications.

### Data analysis

All dogs that received treatment were included in the safety assessments. Only the primary dogs in the European study were included in the efficacy analysis.

The individual dog was the experimental unit. Nematode egg counts per gram of feces were summarized with geometric means by treatment and time-point. Percent efficacy of each treatment was calculated using the formula [(C − T)/C] × 100, where C is the pre-treatment geometric mean and T is the post-treatment geometric mean.

For the European study, fecal egg counts were analyzed across all nematode species, and for each nematode species separately if five or more animals in each treatment group had positive fecal egg counts of a given species prior to treatment.

For the USA study, *T. canis* egg counts were the primary analysis variable. Only sites with two or more evaluable cases in each treatment group were included in the efficacy analysis. Nematode species with five or more animals in each treatment group having positive fecal egg counts prior to treatment were included as secondary analysis variables.

Natural log-transformed fecal egg counts [*log*_*e*_ (*count + *1)] were analyzed using a mixed linear model for repeated measures. The model included the fixed effects of treatment, time, and the treatment by time interaction, and the random effects of clinic, block within clinic, the clinic by treatment interaction, animal within clinic, block and treatment, the interaction of clinic, treatment and time, and error. Geometric mean egg counts for each treatment group at each time point were calculated from the least squares means for log-transformed egg counts. Corresponding back-transformed 95% confidence limits for mean egg counts were constructed. SAS PROC MIXED was used to fit the statistical models and the Kenward-Roger option was used to calculate degrees of freedom for the test-statistics used for pre-treatment *vs* post-treatment comparisons within treatment. All analyses were carried out using SAS/STAT Release 9.4 (SAS Institute, Cary, NC, USA).

## Results

### Patient signalment

Patient signalment is summarized in Table 1.

**Table 1:** Signalment of dogs enrolled as veterinary patients in studies conducted in Europe and the USA to evaluate the efficacy of Simparica Trio™ against natural gastrointestinal nematode infections

Signalment	European study (primary dogs)		USA study	
	Simparica Trio*™* (*n *= 194)	Afox + Milb (*n *= 97)	Simparica Trio*™* (*n *= 120)	Ive + Pyr (*n *= 42)
Purebred	76	39	71	25
Non-purebred	118	58	49	17
Age, Mean (years)	2.7	2.9	0.8	1.3
Age, Range (years)	0.2–15.0	0.2–13.0	0.2–14.0	0.2–9.0
Body weight, Mean (kg)	16.0	17.1	9.1	13.0
Body weight, Range (kg)	2.1–70.0	2.1–51.0	1.5–47.4	1.5–39.8
Male	108	49	65	21
Female	86	48	55	21
Lives mostly indoors	19	8	86	30
Lives mostly outdoors	131	68	3	1
Lives indoors and outdoors	44	21	31	11

*Abbreviations*: Afox, afoxolaner; Milb, milbemycin oxime; Ive, ivermectin; Pyr, pyrantel pamoate

#### European study

Two hundred and ninety-one primary dogs (194 Simparica Trio™; 97 NexGard^®^ Spectra) and 215 supplementary dogs (137 Simparica Trio™; 78 NexGard^®^ Spectra) were enrolled and treated. Patient demographics data was similar between the two groups. The majority of dogs in both treatment groups (≥ 67.5%) spent most of their time outdoors.

#### USA study

One hundred sixty-two dogs (120 Simparica Trio™; 42 Heartgard^®^ Plus) were enrolled and treated. Patient demographics data was similar between the two groups. The majority of dogs in both treatment groups (≥ 71.4%) spent most of their time indoors.

### Safety

In both studies, the abnormal clinical signs observed following treatment were of the type and frequency that would be expected in a general canine population infected with gastrointestinal parasites. Concomitant medications were administered to 1.2% of Simparica Trio™-treated dogs in the European study, and to 71% of Simparica Trio™-treated dogs in the USA study. In the USA study, the most frequently used concomitant medications were various vaccines on 115 occasions and antiparasitics as follows: against tapeworms in 3 dogs (praziquantel), *Giardia* infection in 4 dogs (metronidazole) and coccidia in 7 dogs (sulfadimethoxine). Other medications including analgesics, antibiotics, ophtalamologicals, dermatologicals, nonsteroidal anti-inflammatory drugs, otologicals and corticosteroids, were administered to up to 2 dogs each. In both studies, Simparica Trio^™^ was well tolerated when administered alone or concomitantly with a variety of other medications commonly used in veterinary medicine.

#### European study

There were no serious adverse events and no mortalities. Non-serious adverse events occurred in three Simparica Trio™-treated dogs. These events included emesis on the day of dosing in two dogs (one of which was attributed to motion sickness since it occurred in the car while the dog was being transported from the clinic after dose administration), and clinical signs associated with parvovirus infection in one dog.

#### USA study

There was one serious adverse event in each treatment group. These events included the occurrence of severe clinical signs associated with non-specific gastroenteritis in one Simparica Trio™-treated dog that recovered following supportive care, and the death of one Heartgard® Plus-treated dog infected with parvovirus. Overall, the incidence of post-treatment abnormal clinical signs was nearly identical in the two treatment groups, with 23.3% of Simparica Trio™-treated dogs and 23.8% of Heartgard® Plus-treated dogs experiencing at least one abnormal clinical sign. Abnormal clinical signs occurring in > 2.0% of treated dogs included intestinal infection with parasites other than *T. canis* (10.8% Simparica Trio™ and 7.1% Heartgard® Plus), diarrhea (5.8% Simparica Trio™ and 9.5% Heartgard® Plus), and emesis (2.5% Simparica Trio™ and 11.9% Heartgard® Plus).

### Efficacy

#### European study

One dog that received Simparica Trio™, was not infected with any of the four nematode species of interest; therefore, no pre-treatment data of this animal could be included in the efficacy analysis. Two dogs (one from each treatment group) were withdrawn from the study prior to completion due to owner non-compliance and 18 dogs (13 Simparica Trio^™^ and 5 NexGard® Spectra) were excluded from the post-treatment efficacy analysis due to protocol deviations. Thus, 270 dogs (179 Simparica Trio™ and 91 NexGard® Spectra) were included in the post-treatment efficacy calculation across all nematode species. Two hundred dogs were infected with *T. canis*, 80 with *A. caninum*, 36 with *U. stenocephala*, and 16 with *T. leonina*. Fifty dogs had mixed infections with two or more of these four nematode species.

Post-treatment geometric mean fecal egg counts across all nematode species, and individually for *T. canis*, *A. caninum*, *T. leonina* and *U. stenocephala* relative to pre-treatment counts were significantly (5.87 ≤ *t*_*df*_ ≤ 22.11, where *df*: 12–194, *P *< 0.0001) reduced by 98.9%, 99.0%, 99.0 %, 98.3% and 99.7% in the Simparica Trio™ group, and 98.7%, 99.3%, 97.4%, 99.9%, and 98.3% in the NexGard® Spectra group, respectively (Table 2).

**Table 2:** Efficacy of a single oral dose of Simparica Trio™ against natural nematode infections in dogs presented as veterinary patients in Europe

Nematode species	Sample^a^	Treatment group^b^	*n*	Egg count per gram feces			Efficacy
				Geometric mean	95% CI	% Reduction	Test statistic^c^
Across all species	Pre-treatment	Simparica Trio™	193	93.0	68.4–126.3		
		Afox+Milb	97	108.1	73.2–159.4		
	Post-treatment	Simparica Trio™	179	1.0	0.5–1.7	98.9	*t*_(17.9)_= 22.11, *P *< 0.0001
		Afox+Milb	91	1.4	0.7–2.4	98.7	*t*_(59)_= 16.49, *P *< 0.0001
*T. canis*	Pre-treatment	Simparica Trio™	138	84.5	58.6–121.6	–	
		Afox+Milb	62	104.8	63.0–173.9	–	
	Post-treatment	Simparica Trio™	127	0.8	0.4–1.5	99.0	*t*_(193)_= 19.97, *P* < 0.0001
		Afox+Milb	56	0.7	0.2–1.6	99.3	*t*_(194)_= 14.29, *P *< 0.0001
*T. leonina*	Pre-treatment	Simparica Trio™	9	18.6	6.9–47.9	–	
		Afox+Milb	7	140.0	49.1–395.8	–	
	Post-treatment	Simparica Trio™	9	0.3	− 0.2–1.2	98.3	*t*_(14)_= 6.30, *P* < 0.0001
		Afox+Milb	7	0.1	− 0.4–0.9	99.9	*t*_(14)_= 9.98, *P* < 0.0001
*A. caninum*	Pre-treatment	Simparica Trio™	52	45.3	27.6–74.1	–	
		Afox+Milb	28	49.3	26.7–90.2	–	
	Post-treatment	Simparica Trio™	49	0.5	− 0.1–1.3	99.0	*t*_(28.8)_= 11.21, *P* < 0.0001
		Afox+Milb	28	1.3	0.3–2.8	97.4	*t*_(47.4)_= 8.31, *P* < 0.0001
*U. stenocephala*	Pre-treatment	Simparica Trio™	23	86.9	32.8–227.6	–	
		Afox+Milb	13	106.3	38.5–290.5	–	
	Post-treatment	Simparica Trio™	22	0.3	− 0.5–2.3	99.7	*t*_(20.9)_= 14.60, *P* < 0.0001
		Afox+Milb	13	1.8	0.0–6.5	98.3	*t*_(12)_= 5.87, *P* < 0.0001

^a^Pre-treatment sample collected once between Day-7 and 0; post-treatment sample collected between Day 7 and 14

^b^Single oral administration of Simparica Trio™ on Day 0 to provide dose ranges of 1.2–2.4 mg/kg sarolaner, 24–48 µg/kg moxidectin and 5–10 mg/kg pyrantel (as pamoate salt)

^c^Test statistic and *P*-value for difference (pre-treatment – post-treatment) within group

*Abbreviations*: Afox, afoxolaner; Milb, milbemycin oxime; CI, confidence interval

#### USA study

Efficacy data from eight sites were excluded due to failure of these sites to meet the protocol criteria for minimum number of evaluable cases, which resulted in the exclusion of efficacy data for 33 dogs (26 Simparica Trio™; 7 Heartgard® Plus). From the cases at the remaining sites, five dogs failed to complete the study; four were lost to follow-up (did not return for the Day 10 visit), and one dog died. Efficacy data from 10 dogs were excluded due to various protocol deviations, such as the absence of *T. canis* eggs in the pre-treatment fecal sample, fecal sample collection errors, and dosing errors. Thus, 114 dogs (83 Simparica Trio™; 31 Heartgard® Plus) were included in the efficacy analysis.

Pre-treatment geometric mean *T. canis* fecal egg per gram counts were 273.0 for the Simparica Trio™ group, and 137.7 for the Heartgard® Plus group. Post-treatment mean counts were significantly (*t*_(112)_= 17.18, *P* ≤ 0.0001) reduced by 99.2% in the Simparica Trio™ group, and by 98.6% in the Heartgard® Plus group (Table 3).

**Table 3:** Efficacy of a single oral dose of Simparica Trio™ against natural *Toxocara canis* infections in dogs presented as veterinary patients in the USA

Sample^a^	Treatment group^b^	*n*	Egg count per gram feces		Efficacy	
			Geometric mean	95% CI	% Reduction	Test statistic^c^
Pre-treatment	Simparica Trio™	83	272.98	156.11–476.79	–	–
	Iver+Pyr	31	137.65	62.90–299.85	–	–
Post-treatment	Simparica Trio™	83	2.06	0.75–4.33	99.2	*t*_(112)_= 17.18, *P* < 0.0001
	Iver+Pyr	31	1.89	0.33–5.27	98.6	*t*_(112)_= 9.04, *P *< 0.0001

^a^Pre-treatment sample collected on Day-1 or Day 0 prior to treatment. Post-treatment sample collected between Day 8 and 14

^b^Single oral administration of Simparica Trio™ on Day 0 to provide dose ranges of 1.2–2.4 mg/kg sarolaner, 24–48 µg/kg moxidectin and 5–10 mg/kg pyrantel (as pamoate salt)

^c^Test statistic and *P*-value for difference (pre-treatment – post-treatment) within group

*Abbreviations*: Iver, ivermectin; Pyr, pyrantel pamoate; CI, confidence interval

Other than *T. canis*, only *A. caninum* was present pre-treatment in five or more evaluable cases in each group. In the Simparica Trio™ group, 48 dogs were infected with *A. caninum* and the pre-treatment geometric mean fecal egg count of 1084.3 was reduced by 98.6% post-treatment. In the Heartgard® Plus group 10 dogs were infected with *A. caninum* and the pre-treatment geometric mean fecal egg count of 852.2 was reduced by 74.7%.

## Discussion

Efficacy of Simparica Trio™ was evaluated against *T. canis* in both the European and USA studies based on the reduction in post-treatment geometric mean fecal egg counts compared to pre-treatment egg counts. Efficacy of Simparica Trio™ against *T. canis* was essentially the same, with a 99.0% reduction in the European study and a 99.2% reduction in the USA study. Previously reported studies have shown that Simparica Trio™ provided ≥ 95.2% efficacy against immature adult *T. canis* and ≥ 97.3% efficacy against adult *T. canis* under laboratory conditions [5]. Simparica Trio™ also demonstrated ≥ 98.3% efficacy against *T. leonina*, *A. caninum* and *U. stenocephala* in the European study, and 98.6% efficacy against *A. caninum* in the USA study. These results are in line with the efficacy of Simparica Trio™ demonstrated against induced hookworm and roundworm infections in dogs [5, 6]; thus, the present studies confirm that Simparica Trio™ is also effective under field conditions in dogs of a variety of breeds, ages, and weight ranges, and in dogs most likely infected with varying numbers of ascarids and hookworms at differing developmental stages at the time of treatment.

The efficacy observed in the present field studies is comparable to the results from a recently reported European field study of similar design and methodology [10] in which geometric mean roundworm and hookworm fecal egg counts were reduced by ≥ 97.2% following a single oral dose of afoxolaner + milbemycin oxime, and by ≥ 94.3% following a single oral dose of milbemycin oxime + praziquantel (Milbemax®; Elanco, Greenfield, IN, USA). It was however unexpected that the combination of ivermectin + pyrantel in the present USA study only provided 74.7% efficacy against *A. caninum*. While the reason remains unknown, it has to be noted that these results are based on only a low number (*n *= 10) of dogs.

In addition to the geographical differences, the patient populations were notably different between the European and USA studies. In the European study, the dogs were older (mean age 2.7 years) and the majority (67.5%) spent most of their time outdoors, compared to the USA study where the dogs were younger (mean age 0.8 years) and the majority (71.7%) spent most of their time indoors. In fact, the majority (66.0%) of dogs in the USA study were only 2–3 months of age at the time of treatment. *Toxocara canis* is transmitted trans-placentally *in utero* and to nursing puppies *via* the milk, which results in nearly all puppies being born infected or acquiring infection soon after birth [1, 11]. In adult dogs, patent infections occur from re-activation of somatic larvae and ingestion of infective eggs [1, 11]. Although the definitive route and timing of *T. canis* infection for the dogs in the current studies is unknown, due to their older age and mostly outdoor living, it is likely that the population of dogs in the European study included dogs infected by a variety of routes. In contrast, based on their younger age and mostly indoor living, it is likely that the population of dogs included in the USA study was mostly infected prior to and/or shortly after birth. Despite these patient population differences, the treatments were effective in both studies and provided a ≥ 98.6% reduction in fecal *T. canis* egg counts.

Gastrointestinal nematodes, heartworms, fleas, and ticks are parasites that commonly infect dogs and infections can cause mild to severe clinical disease. Some of these parasites, most notably *T. canis*, are also zoonotic and can cause significant clinical disease in humans. For these reasons, year-round treatment of dogs with antiparasitic agents that provide efficacy against gastrointestinal nematodes, efficacy for prevention of heartworm disease caused by *D. immitis*, and efficacy against fleas and ticks is recommended by the European Scientific Counsel Companion Animal Parasites (ESCCAP) and the Companion Animal Parasite Council (CAPC) of the USA [12, 13]. The three active ingredients in Simparica Trio™ chewable tablets (sarolaner, moxidectin and pyrantel) provide efficacy against these common parasites. The present studies confirm effectiveness of the combination against gastrointestinal parasites in dogs under field conditions, and separate studies have demonstrated its effectiveness in the prevention of canine heartworm disease [14], and its effectiveness against fleas and ticks in dogs under field conditions [15, 16]. The broad-spectrum antiparasitic activity provided by the combination of active ingredients in a single orally administered chewable tablet should allow for ease of administration to better meet recommended treatment and prevention guidelines for these common canine parasites.

## Conclusions

Simparica Trio™ administered once orally to provide the label dose ranges of 1.2–2.4 mg/kg sarolaner, 24–48 µg/kg moxidectin and 5–10 mg/kg pyrantel (as pamoate salt) was effective in the treatment of natural *T. canis*, *T. leonina*, *A. caninum* and *U. stenocephala* infections in dogs presented as veterinary patients in Europe and in the treatment of *T. canis* and *A. caninum* infections in the USA. The novel combination tablet was well tolerated when administered alone and in combination with a variety of medications commonly used in routine veterinary practice.

## Data Availability

All data generated or analysed during this study are provided within the article.
